# Geological processes shaping freshwater biodiversity: a synthesis of global evidence

**DOI:** 10.1002/brv.70135

**Published:** 2026-02-03

**Authors:** Jonathan M. Waters, Christopher P. Burridge, David Craw, James S. Albert

**Affiliations:** ^1^ Department of Zoology University of Otago Dunedin New Zealand; ^2^ School of Natural Sciences University of Tasmania Hobart Tasmania Australia; ^3^ Department of Geology University of Otago Dunedin New Zealand; ^4^ Department of Biology University of Louisiana at Lafayette Lafayette Louisiana USA

**Keywords:** allopatric, alpha‐diversity, drainage, evolution, freshwater, genomics, geomorphology, landscape, river capture, speciation

## Abstract

Recent genomic data highlight the key roles of geological processes in shaping the diversification and biogeography of freshwater lineages. Specifically, physical processes such as tectonic uplift, erosion, glaciation, lake formation, and sea‐level fluctuation contribute extensively to the evolution of biotic diversity within and among drainages. River capture events can simultaneously isolate and merge lineages, with isolation potentially leading to speciation, and secondary contact enhancing alpha diversity within merged river reaches. The increased speciation rates of newly isolated lineages may be countered by their reduced population sizes and increased extinction risks. Knowledge of drainage history is essential for explaining freshwater biodiversity patterns, and also for understanding the drivers and temporal scales of biological evolution. Future interdisciplinary genomic and geological analyses are needed to understand and conserve freshwater biodiversity in a fast‐changing world.

## I. INTRODUCTION

**Fig. 1:**
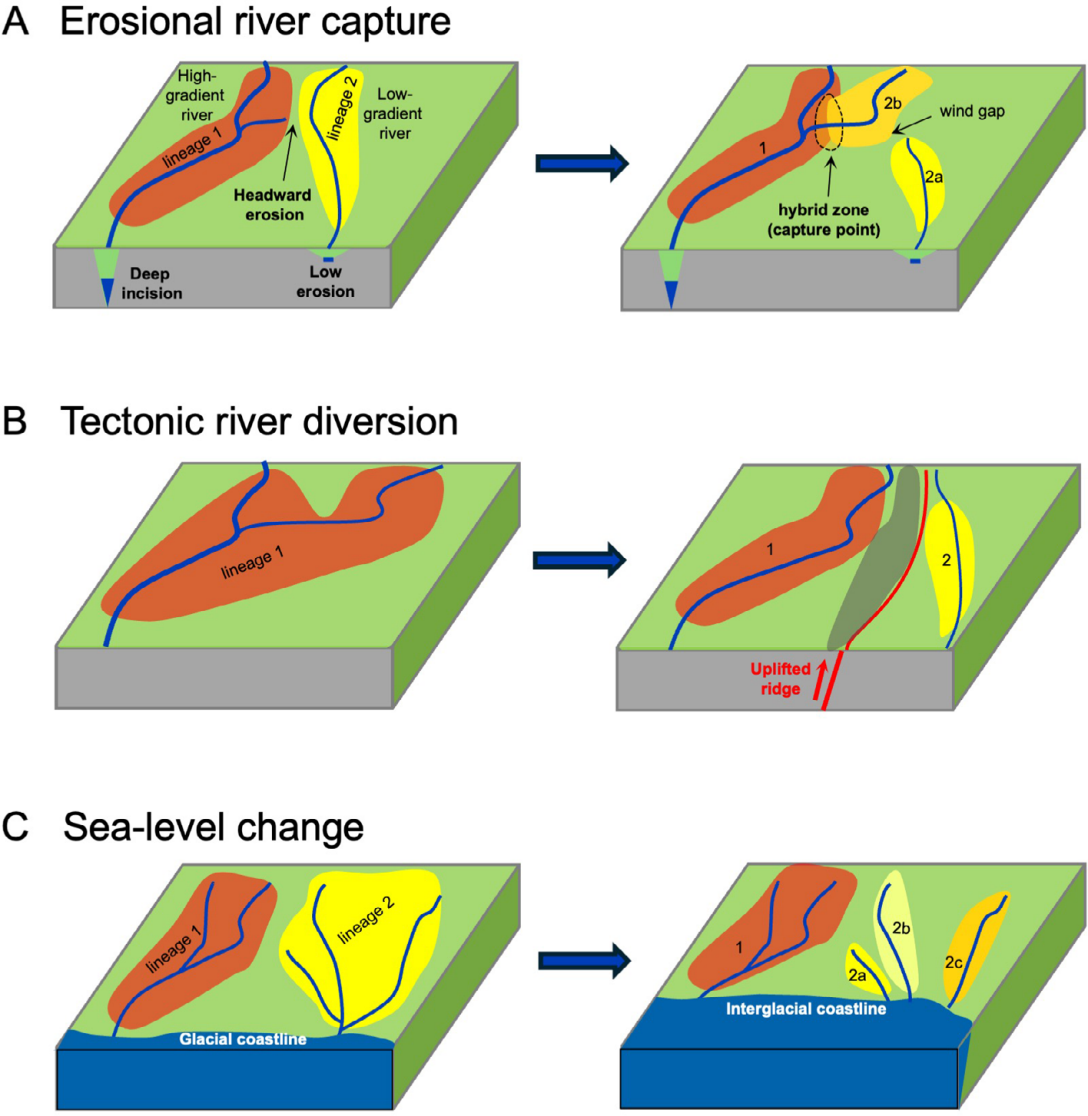
Schematic representation of physical processes shaping evolution of river drainage geometry and freshwater biotic lineages. (A) Erosion‐driven capture of a lowland stream segment by a neighbouring river, leading to freshwater speciation, and the evolution of hybrid zones (Waters *et al*., 2023). (B) Fragmentation of a river segment *via* tectonic uplift, leading to lineage divergence. (C) Climate‐driven isolation and diversification of freshwater‐limited populations due to sea‐level change.

Earth history has long been considered a fundamental driver of biological diversification, with speciation events often linked to geological upheaval, such as the influence of plate tectonics on global volcanism and ocean circulation (Ali & Huber, 2010; Leprieur *et al*., 2016; Shaw & Gillespie, 2016). Freshwater ecosystems are particularly constrained by such physical processes, and these tight links may help to explain the exceptional biodiversity often evident in freshwater habitats [e.g. the ‘freshwater fish paradox’ (Tedesco *et al*., 2012, 2017; Val *et al*., 2022; Hoagstrom, Davenport & Osborne 2025)]. Additionally, by unravelling the drivers of speciation in freshwater ecosystems (Stokes & Perron, 2020), freshwater evolutionary studies have enhanced our understanding of the processes shaping biodiversity more generally. Being constrained by catchment boundaries, freshwater‐limited lineages are subject to the geological processes governing river drainage evolution (Hughes, Schmidt & Finn, 2009; Smith *et al*., 2010; Ruzzante *et al*., 2020; Stokes & Perron, 2020). For example, river segments can be transferred between catchments – a process known as ‘river capture’ – by tectonic uplift, glaciation, and/or erosion (Mayden, 1988; Bishop, 1995; Lyons *et al*., 2020) (Fig. 1). Such events can potentially impact species richness (Stokes & Perron, 2020; Lyons *et al*., 2020), and alter species distributions, initially facilitating range expansion into new catchments (Van Steenberge *et al*., 2020), while also promoting isolation and speciation (‘vicariance’) of previously connected populations (He *et al*., 2024). Conversely, such geological processes can also bring formerly isolated lineages into secondary contact (Barreto *et al*., 2020; Campbell *et al*., 2022; Pierson *et al*., 2024), enhancing local alpha diversity, with introgression potentially fuelling adaptation and diversification (Meier *et al*., 2017; Brauer *et al*., 2023). While the impacts of these geological events are most prominent for strictly freshwater‐limited taxa, drainage shifts can also shape diversification across myriad additional aquatic lineages [e.g. diadromous fishes (Lescak *et al*., 2015); terrestrial insects with aquatic larvae (McCulloch *et al*., 2022)] and non‐aquatic lineages inhabiting floodplain and riparian corridors, including trees, birds, and mammals (Albert, Tagliacollo & Dagosta, 2020; Musher *et al*., 2022; Musher, 2025; Ribas *et al*., 2025). The unique biodiversity arising from these dynamic processes may have particularly high conservation value (Dudgeon *et al*., 2006; Barbarossa *et al*., 2021; Sayer *et al*., 2025).

**Fig. 2:**
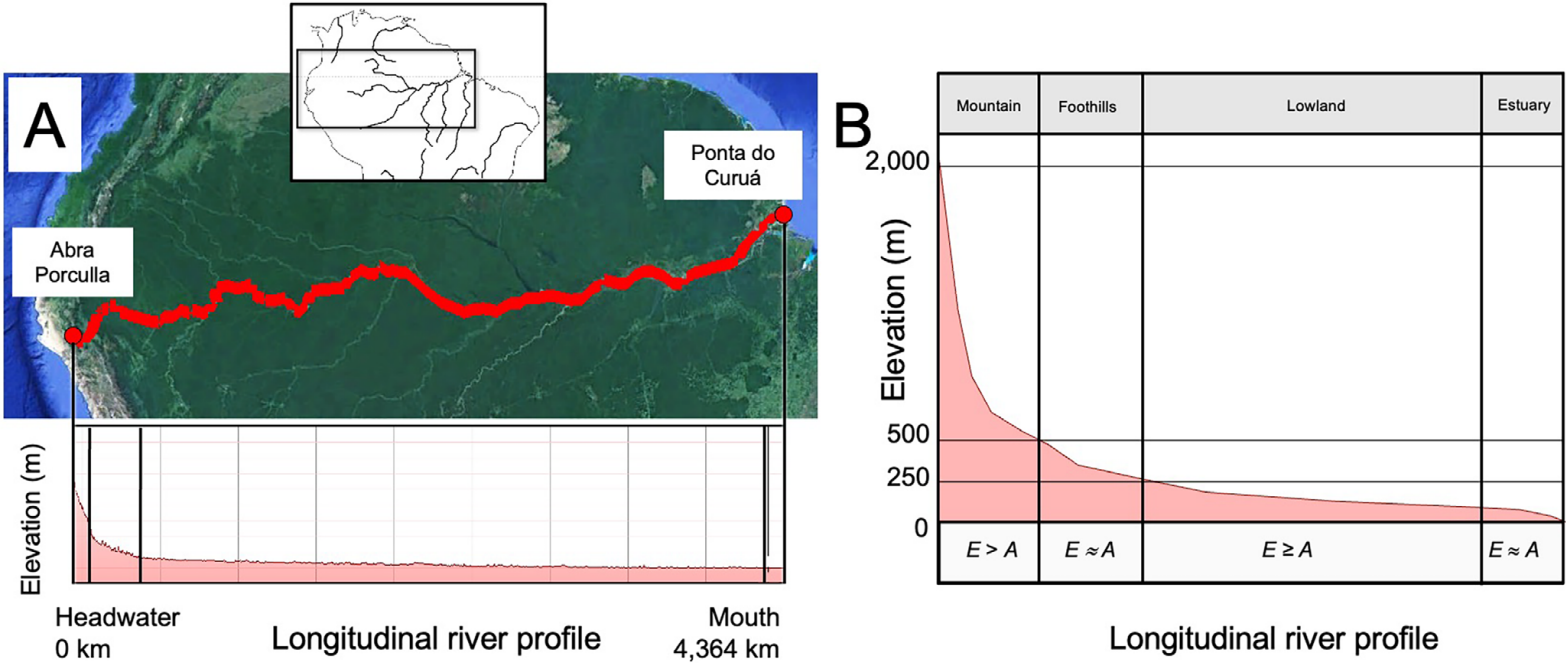
Erosional capture dynamics within major river systems are hypothesised to influence strongly the generation of freshwater biodiversity (e.g. Tagliacollo *et al*., 2015; Val *et al*., 2022; dos Reis *et al*., 2024) and are broadly predictable from river elevation profiles. (A) Longitudinal profile (red line) of the Amazon main‐stem from Abra Porculla in the Peruvian Andes to Ponta do Curuá at Isla Marajo. Images from Google Earth; elevation profile traced using Path tool. Inset depicts location of Google Earth map image. (B) Schematic profile from headwaters (left) to mouth (right), with scale modified to illustrate alternating zones of hydrodynamic processes driving river capture. *E*, rate of erosion from sediment denudation; *A*, rate of avulsion from sediment accumulation. Foothills include alluvial fans, where *E* < *A* when fans are accreting, and *E* > *A* when fans are retreating. Lowlands include floodplain areas where *E* ~ *A* under equilibrium, and non‐floodplain (*terra firma*) areas where usually *E* > *A*. Where *E* > *A*, tributary capture predominates. Where *E* < *A*, distributary (deltaic) capture predominates.

Evidence emerging from diverse environments across the globe, including North America (Kim *et al*., 2023; Stokes *et al*., 2023; Pierson *et al*., 2024), South America (Zemlak *et al*., 2008; Albert & Reis, 2011; Stokes, Goldberg & Perron, 2018; Ruzzante *et al*., 2020; Albert *et al*., 2021; Boschman *et al*., 2023; Cassemiro *et al*., 2023; Ramirez *et al*., 2023), Africa (Goodier *et al*., 2011; Van Steenberge *et al*., 2020), Australia (Waters, Burridge & Craw, 2019), New Zealand (Craw *et al*., 2015), and Japan (Masuda *et al*., 2023), broadly highlights the important role of geological processes in shaping freshwater biodiversity. Drainage connections are continually affected by erosional and depositional processes that can lead to river diversion and reorientation (Fig. 1A), augmented by the effects of climate change. Such drainage alterations can drive freshwater biological diversification over a range of spatial and temporal scales (Burridge *et al*., 2008*a*; Waters, Burridge & Craw, 2020; Van Steenberge *et al*., 2020), some as fine as a few kilometres (Burridge, Craw & Waters, 2006, 2007). Even in geologically stable regions, weathering and erosion over millions of years can promote landscape evolution leading to biological isolation (Waters *et al*., 2019; Kim *et al*., 2023; Stokes *et al*., 2023) (Figs 1 and 2), and it has been hypothesised that such processes may be key drivers of faunal diversification (Val *et al*., 2022). Recently, macroecological simulations (Val *et al*., 2022) have suggested that river capture may be key to explaining the exceptionally high fish diversities of numerous major lowland river systems, including the Amazon (Fig. 2) (Stokes *et al*., 2018), Congo, Mississippi, and Yangtze (Albert *et al*., 2018; Chen *et al*., 2023).

Global tectonic processes lead to widespread surface deformation and landscape evolution that, in turn, may affect freshwater connections and biological evolution (Fig. 1B) (Craw *et al*., 2015). Local or regional uplift and/or subsidence, and accompanying erosion and sedimentary deposition, almost inevitably cause river drainage changes. For instance, numerous freshwater speciation events have been linked to the tectonic formation of mountain ranges such as the Himalaya (Rüber *et al*., 2004; He & Chen, 2006) and Andes (Boschman *et al*., 2023; Cassemiro *et al*., 2023). In addition, continental rifting, such as is currently displayed spectacularly in eastern Africa, leads to zones of subsidence flanked by uplift, and these areas have similarly been focal points for geologically mediated freshwater biological diversification for millions of years (Goodier *et al*., 2011; Danley *et al*., 2012; Van Steenberge *et al*., 2020).

This review aims to highlight the role of diverse abiotic processes shaping the distribution and diversity of freshwater lineages across a variety of spatial and temporal scales (Ruzzante *et al*., 2020; Val *et al*., 2022; Boschman *et al*., 2023). In particular, we synthesise novel insights emerging from combined analyses of geological and freshwater genomic data (Craw *et al*., 2015; Kim *et al*., 2023; Stokes *et al*., 2023). We argue that the tight linkages detected between abiotic and biotic processes provide a strong framework for understanding the evolutionary dynamics of both landscape and biota (e.g. Burridge *et al*., 2008*a*; Craw *et al*., 2008; Lescak *et al*., 2015).

## II. GEOLOGICAL EVIDENCE FOR RIVER DIVERSION EVENTS

**Fig. 3:**
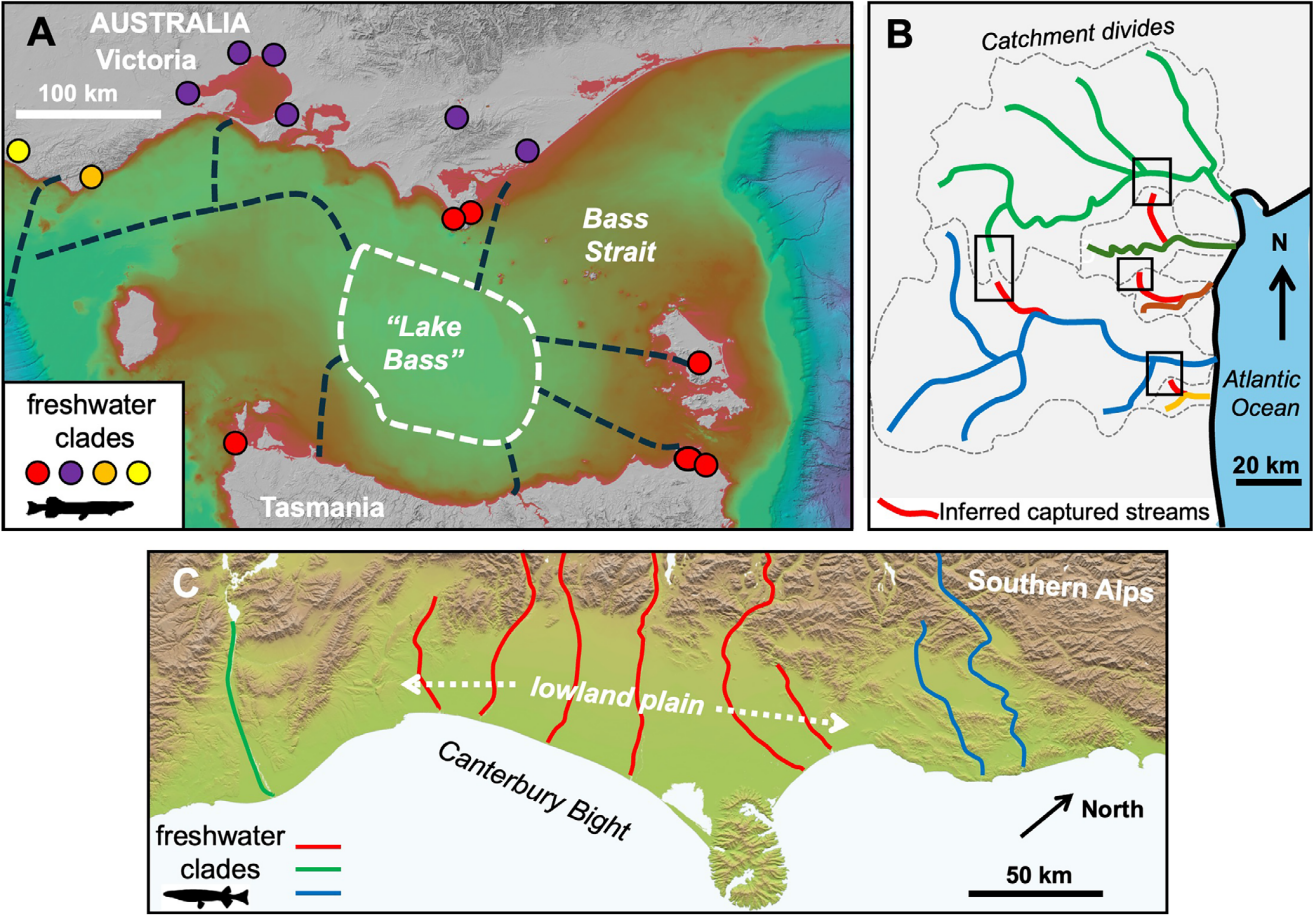
A variety of drainage evolution processes can both connect and isolate freshwater lineages. (A) Palaeodrainage connections (dashed lines) during glacial sea‐level low stands have facilitated dispersal of freshwater‐limited lineages (red) between Tasmania and mainland Australia, with subsequent isolation by sea‐level rise (Horwitz, 1990; Unmack *et al*., 2011, 2012, 2013) (image: www.deepreef.com). (B) A series of upland erosional stream capture events (captured stream tributaries shown in red) explains the dispersal and diversification of *Leporinus* fishes across adjacent coastal drainages in Brazil. Phylogenetic relationships among five river‐specific clades (distinct colours) support this geographic sequence of capture events (Ramirez *et al*., 2023). (C) Periodic lateral migration (white arrows) of river channels across a lowland braid plain (Canterbury Plain) in South Island, New Zealand, have led to regional genetic homogeneity, with genetically shallow ‘plain’ lineages (red) shared widely across this lowland region (Waters & Wallis, 2001; Wallis *et al*., 2001; Apte *et al*., 2007; Waters *et al*., 2015*a*).

**Fig. 4:**
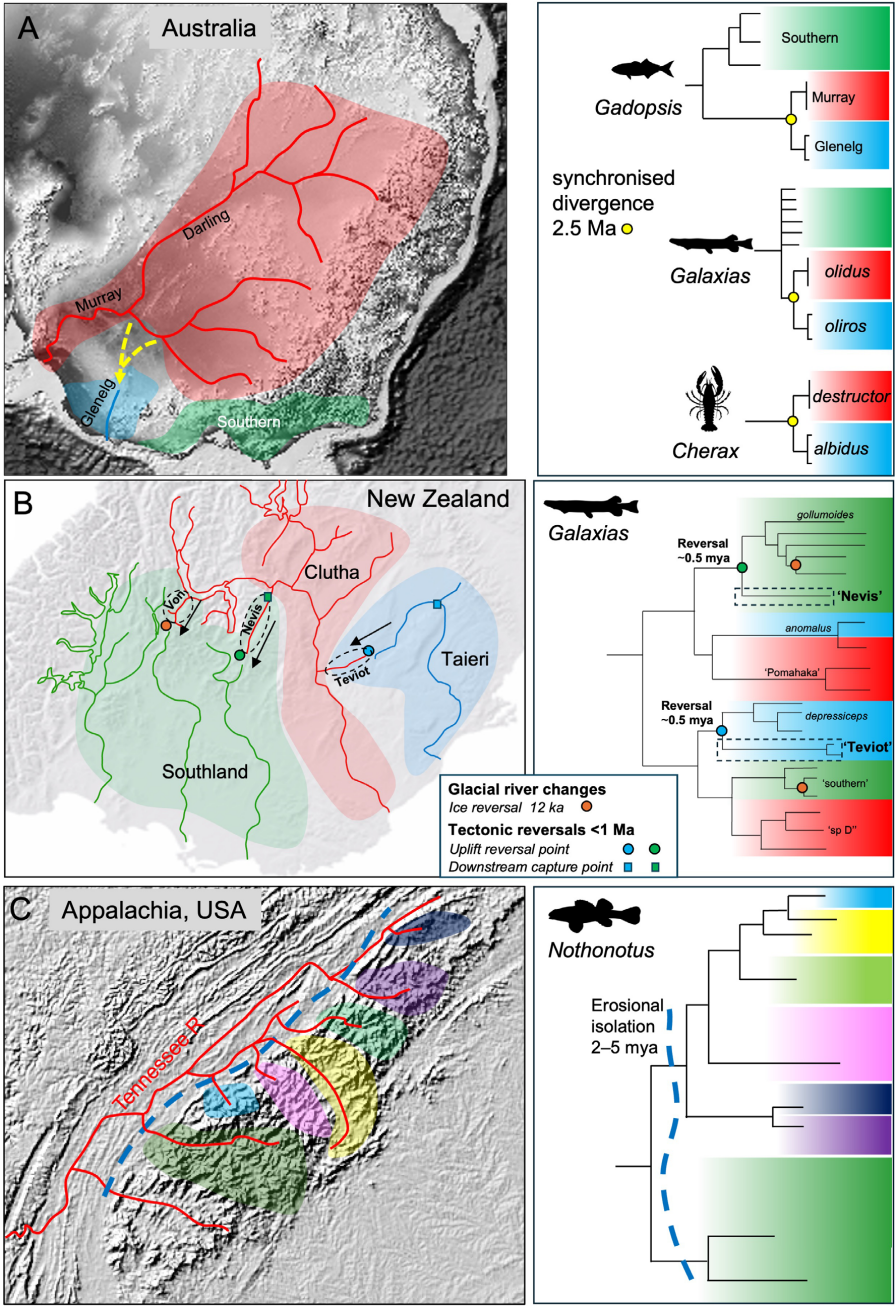
Combinations of biological and geological data can lead to reciprocal illumination of freshwater evolutionary processes. (A) Until the Pliocene, Australia's Murray–Darling river system drained south through what is currently the Glenelg River system. Subsequently, minor Pliocene tectonic uplift forced the Murray–Darling to abandon its former southerly course, instead forming a new outlet further west. This diversion event led to synchronous isolation and speciation events between Murray‐Darling (red) and Glenelg (blue) lineages (Nguyen & Austin, 2005; Adams *et al*., 2014; Hammer *et al*., 2014; Waters *et al*., 2019; Unmack *et al*., 2019; Campbell *et al*., 2024). (B) River reversals (former connections indicated by arrows) have led to speciation (dashed boxes on right) of fish lineages isolated across inland drainage divides in southern New Zealand (Waters *et al*., 2001; Burridge *et al*., 2007; Craw *et al*., 2016; Campbell *et al*., 2022). (C) Pliocene erosion has led to ecological isolation and speciation among Appalachian headwater populations of *Nothonotus* fishes (Stokes *et al*., 2023).

Schematic examples of the spatial geometry of typical river diversions that occur through geological time, contributing to biological diversity of freshwater species, are summarised in Fig. 1. We also highlight diverse examples of such processes, including both geological and biological evidence (Figs 3 and 4). However, finding geological support for specific river capture events can sometimes be difficult, especially for older events obscured by younger events. Evidence can be ranked based on contrasting levels of confidence and frequencies of occurrence. First, some inferred changes are based on (*a*) *direct geological evidence*, such as distinctive river sedimentary rocks and minerals that are exotic to their current catchment, and/or associated indicators of changes in sedimentary transport directions (Fig. 4). Secondly, some inferences include (*b*) *indicative geological support*, such as low drainage divides and barbed stream geometry (Fig. 1A). Thirdly, some systems include (*c*) *permissive geological support*, in which river diversion events in general may be plausible conclusions based on regional geological history, with such examples comprising the bulk of cited evidence (e.g. Waters, Lintermans & White, 1994; Taylor, Stamford & Baxter, 2003; Sousa‐Santos, Collares‐Pereira & Almada, 2006; Hughes *et al*., 2009; Tagliacollo *et al*., 2015; Musher *et al*., 2022). For cases involving (*b*) *indicative geological support*, biological observations can be of considerable value to geologists trying to understand the topographic evolution of a region. In particular, genetically inferred biotic relationships can provide direct evidence for river diversion, defining which rivers were involved and providing estimates of timing (Rüber *et al*., 2004; He & Chen, 2006; Craw *et al*., 2008, 2015). While the absence of *direct geological support* for a river capture event can be perceived as a weakness (Bishop, 1995; Waters & Craw, 2006), several recent genetic analyses have generated important novel evolutionary hypotheses that provide fertile ground for geological analysis (Van Steenberge *et al*., 2020; Ramirez *et al*., 2023; dos Reis *et al*., 2024).

## III. GEOLOGICAL PROCESSES SHAPING FRESHWATER BIODIVERSITY

Our synthesis of global data reveals that ongoing geological processes (e.g. tectonics, glaciation, erosion, sediment deposition) can affect the evolution of freshwater lineages across a range of spatial and temporal scales. Erosional and depositional processes in topographically complex landscapes can constrain diversification of freshwater lineages (topographically distinct streams; gorge formation; Fig. 1A). By contrast, tectonic uplift processes have underpinned lineage divergences *via* drainage divide migration and mountain building in the context of global plate tectonics (Fig. 1B). While geological evidence of river reorientation events is often particularly striking in mountainous regions (Fig. 4B), there are also numerous examples of lowland river drainage evolution resulting in the isolation and merging of lowland fish populations (Fig. 3). It should be noted that many of these processes can be ongoing, with potential for more recent events to ‘overwrite’ the geological signatures of older events. Below we review in more detail the nature of such geological processes and their impacts on the evolution of freshwater biotas.

### (1) Tectonic processes

**Fig. 5:**
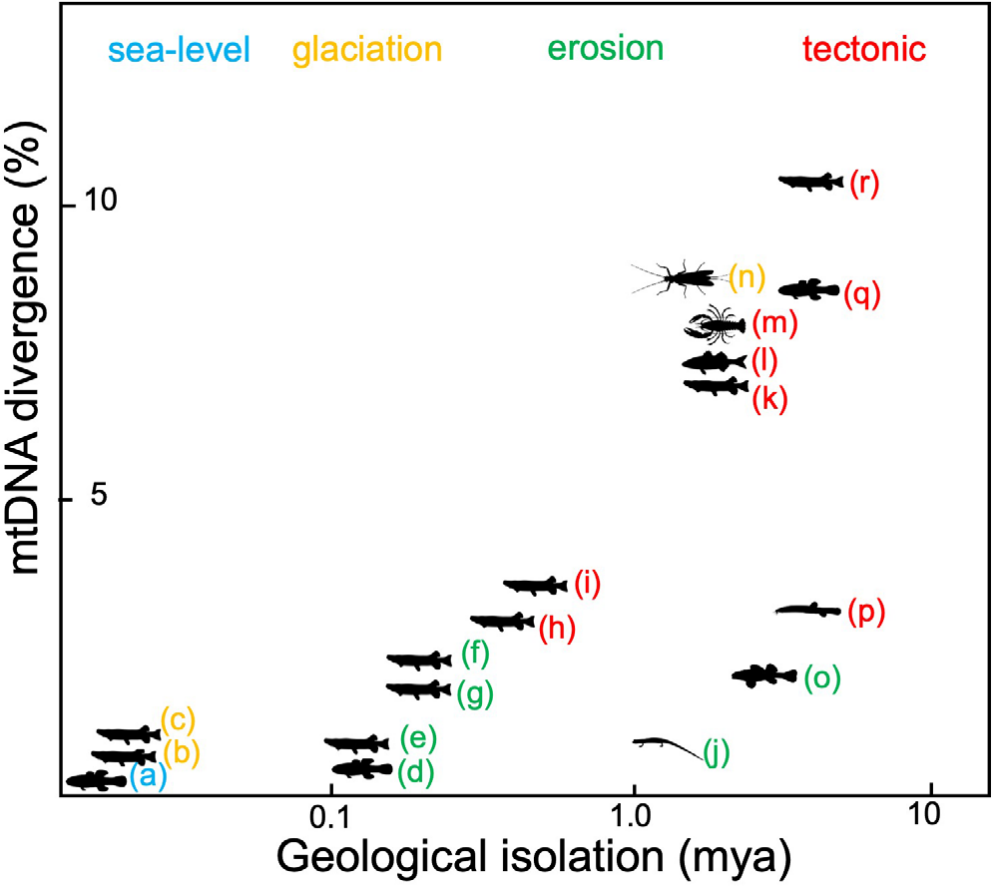
A synthesis of relationships between geological time [log scale; million years ago (Ma)] and mitochondrial DNA sequence divergence among freshwater lineages. Dates of freshwater isolation events are inferred from diverse geological analyses (references below), and associated genetic divergences are assessed using mitochondrial DNA sequence comparisons. Divergence events are colour‐coded by geological process. (a) Pelorus *versus* Kaituna (South Island) *Gobiomorphus* fishes (Waters *et al*., 2007); (b) Clarence *versus* Wairau (South Island) *Galaxias* fishes (Burridge *et al*., 2006); (c) Von *versus* Oreti (South Island) *Galaxias* (Burridge *et al*., 2007); Kaituna *versus* Wairau (South Island) (d) *Gobiomorphus* and (e) *Galaxias* (Waters *et al*., 2007); (f, g) Oreti *versus* Mararoa (South Island) *Galaxias* (Burridge *et al*., 2008*b*); (h) Nevis *versus* Mataura (South Island) *Galaxias* (Waters *et al*., 2001); (i) Teviot *versus* Taieri (South Island) *Galaxias* (Waters *et al*., 2015*b*); (j) New *versus* Roanoke (Appalachian) *Eurycea* salamanders (Kozak *et al*. 2006); (k) Murray *versus* Glenelg (Australia) *Galaxias* (Adams *et al*., 2014); (l) Murray *versus* Glenelg (Australia) *Gadopsis* fishes (Hammer *et al*., 2014); (m) Murray *versus* Glenelg (Australia) *Cherax* crayfish (Nguyen & Austin, 2005); (n) northern *versus* southern (South Island) *Halticoperla* stoneflies (Wallis *et al*., 2016); (o) northern *versus* southern Appalachian *Nothonotus* fishes (Stokes *et al*., 2023); (p) Iberian *versus* African *Cobitis* fishes (Doadrio & Perdices, 2005); (q) eastern *versus* western South Island *Gobiomorphus* (Smith *et al*., 2005); (r) eastern *versus* western South Island *Galaxias* (Burridge *et al*., 2008*a*). Images from PhyloPic.org.

Particularly strong inferences of linked riverine and biotic evolution can now be generated by addressing ongoing tectonic processes across a range of spatial and temporal scales (Fig. 1B) (Craw *et al*., 2015, 2019). While uplift has played a particularly dramatic role shaping drainage evolution in numerous regions (Craw *et al*., 2015; Conde‐Saldaña *et al*., 2025), drainage patterns can also be heavily impacted by subsidence (Van Steenberge *et al*., 2020; Waters *et al*., 2020). Tectonic uplift has been crucial in the late Pliocene isolation and speciation of lowland vertebrate and invertebrate lineages of southeastern Australia (Fig. 4A) (Waters *et al*., 2019), and in the late‐Pleistocene diversification of freshwater fishes in southern New Zealand (Fig. 4B) (Waters *et al*., 2001, 2007; Burridge *et al*., 2008*a*; Craw *et al*., 2007*a*, 2015, 2016). The well‐defined geochronology of these regions has also been instrumental in calibrating and understanding the dynamics of molecular clocks for freshwater fishes (Waters *et al*., 2019, 2020; Fig. 5). In New Zealand, the oldest recognisable genetic divergence arose when the rapid [<5 million years ago (Ma)] rise of a 3,000 m high mountain range, the Southern Alps caused separation of freshwater‐limited galaxiid and eleotrid fishes into distinct eastern and western clades. Additionally, more recent and localised divergence *within* these deep clades can similarly be explained by ongoing local tectonic uplift processes (e.g. Figure 4B) (Burridge *et al*., 2008*a*; Craw *et al*., 2015, 2019). Causal links between gradual tectonic uplift and the deep‐time phylogenetic diversification of freshwater fishes have recently been inferred in South America (Albert *et al*., 2020, 2021). Specifically, the striking diversity and biogeographic structure of Amazonian (Fig. 2) fishes is thought to reflect gradual landscape changes influenced by 80 million years of continuous uplift of the Andes (Boschman *et al*., 2023).

### (2) Erosional and depositional processes

River capture *via* headward erosion of one valley into a neighbouring catchment (Fig. 1A) is a commonly cited driver of geologically driven diversification in freshwater lineages (Bishop, 1995; Albert *et al*., 2018, 2020; He *et al*., 2024). These erosional capture events have been most frequent in small to medium sized lowland catchments (Albert *et al*., 2018), probably because these landscapes typically have softer substrates and are less incised relative to upland regions. Consequently, considerable freshwater faunal diversification can occur even in tectonically quiescent areas such as eastern South America (Ramirez *et al*., 2023) (Fig. 2) and the slopes of Appalachia of eastern North America (Fig. 4C) (Pierson *et al*., 2024). Major erosional river capture events in large catchments in upland areas are rarer (Albert *et al*., 2018), but among the most spectacular examples is the Miocene capture of the Tsangpo River of Tibet by headward erosion through the Himalaya by the Brahmaputra River, creating the deepest gorge in the world and isolating several species of freshwater fish (Rüber *et al*., 2004; He & Chen, 2006; Craw *et al*., 2019). Erosion is also inevitably followed by deposition of eroded material, potentially triggering subsequent river diversions. Erosional and depositional processes are particularly likely to lead to river diversions when there are contrasts in erosion rates across a drainage divide due to differences in stream gradient, rock type, or climate (He *et al*., 2024). These contrasting conditions are commonly inter‐related, and the capture of the Tsangpo by the Brahmaputra River reflects a combination of all three factors.

Changes to river connectivity by erosion and deposition are not restricted to headwaters. In lowland settings, braided rivers or deltas can have frequent (decadal–millennial) changes in courses, with lateral migration of channels on scales of kilometres or more (Fig. 3C) (Waters & Wallis, 2001; Wallis *et al*., 2001). These dynamic floodplains are characterised by low topography and absence of drainage divides, and thus erosional and depositional processes cause lateral migration of rivers, and potential connections between neighbouring drainage systems, sometimes leading to increased genetic homogeneity among populations (Fig. 3C). An excellent example of lowland drainage evolution is currently found in the Casiquiare river of South America, which actively flows across the watershed divide between the Amazon and Orinoco rivers following a combination of erosion and sedimentary deposition (Winemiller *et al*., 2008; Stokes *et al*., 2018). Crucially, recent macroecological analyses suggest that such river diversions in lowland settings (Fig. 2) may be particularly important drivers of freshwater biodiversification (Tagliacollo *et al*., 2015; Val *et al*., 2022).

Recent studies suggest that erosion can also play a major role in shaping freshwater biodiversity patterns within river catchments (Conde‐Saldaña *et al*., 2025). Most dramatically, the erosional removal of ecologically important (habitat‐forming) rock types is suggested to have isolated headwater clades of *Nothonotus* (Fig. 4C) (Stokes *et al*., 2023) and *Etheostoma* (Kim *et al*., 2023) darters. Erosion can also lead to the formation of ecological barriers within river systems, with gorge formation (e.g. high‐gradient stream sections unsuitable for fishes preferring low‐gradient habitats) contributing to the spatial and genetic structuring of fish lineages within river systems (Waters *et al*., 2001, 2015*a*; Burridge *et al*., 2007).

### (3) Glacier‐driven river reorientation

Glacial–interglacial cycles during the Pleistocene have contributed globally to climate‐induced range shifts for numerous taxa, resulting in both lineage divergence and secondary contact (Hewitt, 2000). Additionally, in freshwater systems, glaciation has influenced species distributions *via* drainage reorganisation (Shugar *et al*., 2017), displacing taxa across drainage divides. Arguably the most elegant examples of these processes globally are linked to the deglaciation of the Patagonian ice sheet (Turner *et al*., 2005; Thorndycraft *et al*., 2019; Benito & Thorndycraft, 2020). Specifically, catastrophic drainage shifts following deglaciation have driven repeated redistribution of fish lineages across the Andean main divide in Patagonia (Ruzzante *et al*., 2008, 2020; Zemlak *et al*., 2008, 2010, 2011; Vera‐Escalona *et al*., 2015). Here, glacial ice periodically filled and modified pre‐existing low‐relief corridors through the mountains that were formed by older river capture events. Similar examples are seen over smaller spatial scales in New Zealand, wherein glacial ice has dammed headwaters, locally reversing drainages *via* lake formation, and promoting the movement of fish among adjacent drainages (Fig. 4B) (Burridge *et al*., 2006, 2007, 2008*a*,*b*). In lowland rivers, Pleistocene cold‐climate settings can also lead to enhanced erosion and re‐deposition of extensive downstream gravel deposits, choking pre‐existing valleys and driving drainage reorientation (Burridge *et al*., 2008*b*). Since such lowland gravel deposits are often incohesive, they can be subsequently re‐eroded and re‐deposited, causing further local drainage reorientation.

### (4) Sea‐level fluctuations

Sea‐level change associated with Pleistocene glacial–interglacial cycles has contributed extensively to the dispersal and distributions of freshwater‐limited fauna among currently isolated lowland drainage systems (De Bruyn & Mather, 2007; Ruzzante *et al*., 2011; Unmack *et al*., 2011, 2012; Swartz *et al*., 2014; Todd *et al*., 2014; Wendt *et al*., 2019; Abreu *et al*., 2020; Campbell *et al*., 2024). In such cases, continental shelf width may play a key role influencing the extent of connectivity between adjacent coastal rivers during low‐sea‐level stands (Figs 1C and 3C) (Unmack *et al*., 2013). Indeed, global biogeographic analyses have yielded consistently strong links between lowland Last Glacial Maximum (LGM) palaeodrainage connections and contemporary freshwater fish distributions (Dias *et al*., 2014). Pleistocene sea‐level change has also influenced floodplain erosional/depositional cycles (Irion *et al*., 2009), with these landscape dynamics in turn influencing lowland freshwater biological connectivity (Val *et al*., 2022).

On deeper geological timeframes, major marine transgressions have been proposed as possible explanations for the origins of numerous marine‐derived freshwater radiations in the Amazon [Miocene marine incursion hypothesis (Webb, 1995; Lovejoy, Bermingham & Martin, 1998; Lovejoy, Albert & Crampton, 2006; Hoorn *et al*., 2010; Bloom & Lovejoy, 2017)] and elsewhere (Yang, Hou & Li, 2013). Such ancient incursions could also have contributed to the generation of large‐scale freshwater biodiversity gradients (Oberdorff *et al*., 2019).

### (5) Lake formation

As noted above, drainage evolution events can be mediated by a wide range of geological processes (e.g. tectonics, erosion, deposition). Lakes, which are home to some of the world's most spectacular adaptive radiations, highlight the biological impacts of these diverse physical processes. Specifically, the tectonic (Danley *et al*., 2012; Lescak *et al*., 2015), glacial (Bernatchez & Wilson, 1998; Schluter & Conte, 2009), and volcanic (Barluenga *et al*., 2006) processes that can individually govern lake formation play crucial roles setting the stage for (if not driving) such freshwater biological evolution. The documented timeframes of such physical processes can also enable researchers to quantify the pace of associated biological evolution (Bernatchez & Wilson, 1998; Lescak *et al*., 2015; Barluenga *et al*., 2006).

Lake formation may also perform a crucial role in river diversion events. For instance, the lowland diversion of the expansive Murray–Darling river complex of eastern Australia (Fig. 4A) involved the formation of a lake that was dammed by uplifting hills and spilt into a new drainage, with a period of freshwater biological interchange between two catchments (Waters *et al*., 2019). While such lakes are temporary on geological timescales, they can form wet connections across the old divides and permit ongoing connections for freshwater biota (Burridge *et al*., 2008*b*; Pierson *et al*., 2024).

In terms of their effect on freshwater biodiversity, large (>10^4^ km^2^), geologically persistent (>10^5^ years) tectonic and glacial lakes can be contrasted with smaller and geologically ephemeral lakes of volcanic, landslide, shoreline, floodplain or other fluvial origins. Adaptive radiations of freshwater fishes are generally limited to large, ancient lakes, mostly of tectonic origin, with less‐diverse radiations in lakes of glacial origin (Seehausen & Wagner, 2014; Albert *et al*., 2020). The relatively few fish species in young lakes proves the rule (Martin & Wainwright, 2013; Gillespie *et al*., 2020).

## IV. FRESHWATER BIOLOGICAL EVIDENCE FOR DRAINAGE EVOLUTION

Understanding how river capture affects the evolutionary diversification of freshwater fishes emerged gradually in the 20th century (Woodworth, 1894; Eigenmann, 1906, 1912, 1921; Hubbs, 1931; Hora, 1937; Wheeler & Cook, 1954; Tsai & Raney, 1974; Hocutt, 1979; Bailey & Smith, 1981; Smith, 1981; Mayden, 1988; Skelton *et al*., 1994). Early studies investigating the effects of geological processes on freshwater taxa relied primarily on species distributional data, sometimes finding biogeographic evidence for past headwater connections between currently isolated drainages (e.g. Mayden, 1988; Bãnãrescu, 1990). The subsequent widespread application of molecular approaches provided greater sensitivity and rigour, by enabling tests of whether intraspecific phylogeographic relationships and lineage divergence times were consistent with *a priori* geologically based hypotheses (Waters *et al*., 2001; Near & Keck, 2005; Kozak, Blaine & Larson, 2006; Johnson, 2007). Furthermore, phylogeographic studies have also identified candidate areas of drainage evolution that were not previously geologically recognised (Gollmann *et al*., 1997; Echelle & Echelle, 1998; Strange, 1998; Engelbrecht *et al*., 2000; Hughes *et al*., 2009). Most recently, genome‐wide analyses have facilitated more rigorous phylogenetic reconstructions of freshwater lineage history (Boschman *et al*., 2023; Cassemiro *et al*., 2023; Masuda *et al*., 2023; Ramirez *et al*., 2023; Stokes *et al*., 2023; Pierson *et al*., 2024). Regardless, there are different ways in which such genetic analyses might support the role of geological processes in freshwater species diversification, as outlined below.

### (1) Phylogeographic anomalies across drainage divides

Freshwater lineages are typically structured by catchment and subcatchment boundaries (Campbell *et al*., 2022; Stokes *et al*., 2023). Therefore, the seemingly anomalous distribution of clades *across* major inland drainage divides can provide key evidence of past drainage evolution (Burridge *et al*., 2006, 2007; Craw *et al*., 2015; Waters *et al*., 2015*b*; Campbell *et al*., 2024). While such redistribution of lineages among catchments could alternatively be explained by anthropogenic processes (Campbell *et al*., 2022) or periodic wet connections among drainages [e.g. swampy divides (Craw *et al*., 2007*a*; Burridge *et al*., 2008*b*)], several studies have uncovered phylogeographic evidence for biotic connectivity alongside direct geological evidence of headwater capture (Waters *et al*., 2001; Pierson *et al*., 2024; Burridge *et al*., 2006), and such scenarios can be formally tested on chronological grounds (Burridge *et al*., 2008*b*).

### (2) Patterns across co‐distributed lineages

The detection of concordant spatial and temporal differentiation across *multiple* co‐distributed taxa can increase confidence in the drivers of freshwater evolution. Specifically, abrupt shifts in drainage geometry (e.g. river reversal) can simultaneously fragment populations of multiple co‐distributed species, leading to concordant phylogeographic patterns among distinct taxa (Waters *et al*., 2019). In central New Zealand, two unrelated freshwater genera show genetic signatures of recent, simultaneous vicariance (Craw & Waters, 2007; Waters *et al*., 2007). Likewise, the detection of similar and deep phylogenetic divergence patterns across unrelated fish genera potentially linked to the ancient Tsangpo river capture implies a shared multispecies vicariant history in the Tibet region (Rüber *et al*., 2004; He & Chen, 2006). By contrast, conflicting phylogeographic patterns observed across co‐distributed species might reflect their ecological or physiological differences (e.g. habitat preferences, dispersal ability, salinity tolerance), which can also be considered *a priori*. For example, species inhabiting main river channels may be more likely to show signatures of river capture (Fig. 1A) than do swamp‐dwelling taxa that can disperse between catchments more readily (Burridge *et al*., 2008*b*). Similarly, cases in which co‐distributed taxa show spatially concordant but temporally dissonant phylogeographies may reflect their intrinsic biological differences (Tagliacollo *et al*., 2017; Thomaz & Knowles, 2020). Alternatively, discordant phylogeographic patterns across currently co‐distributed taxa could reflect differences in lineage age or past distributions (Burridge *et al*., 2006).

## V. THE ROLE OF GEOLOGICAL HETEROGENEITY IN BIOLOGICAL EVOLUTION

Emerging data from several regions suggest that geological diversity (i.e. contrasting rock types) can contribute to freshwater biological diversity. Such patterns could be mediated through taxa adapting to geologically determined habitat features (Kim *et al*., 2023; Stokes *et al*., 2023), or alternatively by geology influencing the likelihood of physical isolation among populations (Waters *et al*., 2015*a*). For example, in the case of some Appalachian fishes, researchers have proposed that adaptation to specific rocky substrates has contributed to the isolation and divergence of lineages across these different geological zones (Fig. 4C; Kim *et al*., 2023; Stokes *et al*., 2023). Waters *et al*. (2015*a*) used a comparative approach to show that different landforms, associated with distinct rock types (schist forming rocky gorges; greywacke forming gravel beds), are linked to contrasting patterns of population‐genetic structure, with markedly higher within‐river differentiation detected in species occupying schist habitats. Such geologically based biological patterns could involve either ‘adaptive’ or ‘neutral’ evolutionary processes (or both): striking genetic structure associated with distinct rock types (Stokes *et al*., 2023) could reflect a combination of ecological (niche‐driven) and physical isolation (Waters *et al*., 2015*a*). Strong ‘tributary‐level’ divergence of freshwater populations (McCulloch *et al*., 2022) also may be maintained by ‘priority effects’, wherein established lineages/populations suppress the genetic contributions of dispersing individuals (Burridge *et al*., 2006; Waters, Fraser & Hewitt, 2013).

## VI. TIMEFRAMES OF FRESHWATER EVOLUTION

Molecular clock approaches have allowed researchers to discriminate between alternative *a priori* abiotic explanations for the origin and diversification of freshwater lineages (Craw *et al*., 2008; Boschman *et al*., 2023; Cassemiro *et al*., 2023). Broadly, there are numerous hypotheses of both climate‐ and tectonic‐driven diversification in freshwater fishes over deep time, from their Palaeozoic origins (Cavin, 2017), Cretaceous and Paleogene radiations (López‐Fernández & Albert, 2011; Nakatani *et al*., 2011; Cavin, Forey & Lécuyer, 2007; Capobianco & Friedman, 2019; Lavoué, 2020), through to the Neogene formation of modern faunas (Albert & Carvalho, 2011; Bloom & Lovejoy, 2017; Melo *et al*., 2021; Conde‐Saldaña *et al*., 2025). Relevant tectonic processes include ancient supercontinent cycles, tectonics associated with orogenies, and short‐scale (0.1–1.0 Ma) geological processes associated with river capture and local volcanics. Similarly, key climate‐driven processes influencing freshwater biotas encompass ancient greenhouse–icehouse oscillations, and more recent Pleistocene and Holocene (Shugar *et al*., 2017) climate fluctuations.

In some cases, the use of geologically dated river diversion events has enabled researchers to quantify rates of biological and molecular evolution better (Craw *et al*., 2007*b*; Waters *et al*., 2007; Burridge *et al*., 2008*a*). This approach requires linking of freshwater biotic isolation events to specific dateable landscape changes. Well‐dated drainage evolution events are typically Miocene to Holocene in age (~5 Ma to <10 ka) (Burridge *et al*., 2008*a*; Waters *et al*., 2019; Doadrio & Perdices, 2005). Although such processes have undoubtedly been ongoing throughout evolutionary history, the geological record of older landscape events and processes isolating freshwater biota typically becomes progressively obscured by subsequent events. Nevertheless, in some cases older diversification events (e.g. tens of million years ago) can be linked to regional geological changes (Albert *et al*., 2020; Boschman *et al*., 2023).

The significance of geologically dated freshwater isolation events for our understanding of molecular rates of evolution is demonstrated by the calibration of mitochondrial DNA (mtDNA) clocks for freshwater fish using independently dated young geological events (e.g. Figs 4 and 5) (Craw *et al*., 2008; Waters *et al*., 2019, 2020). These temporal calibrations have suggested that issues of time dependency—contrasting rates inferred over different timescales—can potentially complicate molecular dating across different timescales (Burridge *et al*., 2008*a*). However, it appears that calibrated divergence rates become relatively linear over deeper timescales (>1 Ma) (Burridge *et al*., 2008*a*; Waters *et al*., 2019), thus indicating that molecular approaches have strong potential for dating the effects of older tectonic processes on freshwater biotic evolution [e.g. continental drift (Burridge *et al*., 2012; Matschiner *et al*., 2020)], provided that appropriate calibration points are chosen, and that any issues of sequence saturation are carefully addressed (Phillips *et al*., 2013). While previous rate calibration studies have largely relied on non‐recombining mtDNA sequences (Burridge *et al*., 2008*a*), such single‐locus approaches can be hampered by issues such as incomplete lineage sorting and introgression (Campbell *et al*., 2022). Moving forward, the widespread use of high‐throughput sequencing offers new opportunities for temporal calibration of genome‐wide sequence evolution (Stokes *et al*., 2023).

Our synthesis of geologically dated freshwater divergence events suggests that different types of geological processes appear to be associated with different levels of genetic divergence (Fig. 5). However, this finding may reflect the extent to which physical evidence of different types of geological upheaval is preserved. Specifically, the impacts of climate‐driven change (glaciation, sea‐level change) often involve relatively shallow genetic splits. Indeed, most evidence for drainage changes associated with glaciation is preserved in the Late Pleistocene record (Burridge *et al*., 2006, 2007; Pierson *et al*., 2024). Nevertheless, genetic records of older glacial/interglacial events can persist, such as in galaxiid fish (Craw *et al*., 2008; Zemlak *et al*., 2008, 2010) (0.2–1.5 Ma), and even to ~2 Ma for some freshwater insect taxa (Wallis *et al*., 2016; Craw, Waters & Burridge, 2017). Interestingly, the geological records of relatively old tectonic and erosional processes can be preserved even over fine spatial scales. Local examples of ancient divergence include highly divergent stonefly lineages isolated in neighbouring streams (kilometre scales) within a single mountain range (McCulloch *et al*., 2022), and similar erosional isolation of neighbouring stream populations of fishes in the tectonically quiescent Appalachian region (Fig. 4C) (Stokes *et al*., 2023).

## VII. INTROGRESSION

While previous research has focused on the role of drainage evolution in *isolating* lineages, new evidence also points to a key role for geological processes in *reuniting* lineages (Fig. 1A) (Zemlak *et al*., 2008; Pierson *et al*., 2024). Analysis of the hybrid zones resulting from these processes can shed light on the extent to which reproductive barriers have evolved among the formerly isolated lineages, and can thus enhance our understanding of biological speciation processes. Extensive hybridisation between freshwater lineages can present challenges for systematic studies, with several recent studies having detected extensive mitonuclear discordance (Wallis *et al*., 2017), including cases of mitochondrial ‘capture’ (Perea *et al*., 2016; Unmack *et al*., 2017 Wallis *et al*., 2017; Campbell *et al*., 2022), perhaps providing genomic parallels to the captures of rivers themselves. Such introgression events (Barreto *et al*., 2020; Waters, Campbell & Dutoit, 2023) have potential to enhance adaptive capacity of otherwise isolated freshwater lineages, as they can expedite the acquisition of advantageous genetic variants (Brauer *et al*., 2023).

## VIII. CONCLUSIONS

(1) Emerging data from several regions point to astonishingly tight links between freshwater geological and biological processes. It is thus essential that freshwater biodiversity studies increasingly incorporate physical frameworks to understand these links better, with analyses of biotic and abiotic data directly informing one another. For example, given that relatively few river capture events have been documented from both biological and geological perspectives, more work is urgently needed to clarify the links between these physical and biological processes.
(2) Recent studies highlight the underappreciated potential for using freshwater geological isolation events to calibrate rates of biological evolution (Barluenga *et al*., 2006; Burridge *et al*., 2008*a*; Lescak *et al*., 2015). However, there remains an urgent need for improved dating of physical processes to elucidate better the temporal scale of biological and genomic change. Currently, this exciting potential for freshwater‐biogeographic calibrations of evolutionary rates remains relatively unfulfilled (Fig. 5).
(3) Genetic approaches currently represent an under‐appreciated tool for inferring geological processes. In regions where the geological record itself remains obscure or has been largely lost through subsequent erosion, genomic tools promise to yield new insights into ancient geological processes. In these settings, biological data can help to distinguish among various scenarios that have been postulated from geological observations. Molecular clocks (Fig. 5) have potential to inform future geological analyses by calibrating rates of geological change that may be difficult to quantify by other means.
(4) Can river capture processes explain the astonishing freshwater biodiversity found in numerous large lowland river settings? There is considerable scope for macroecological studies to assess further how past and present hydrological processes have interacted with organismal traits to influence large‐scale freshwater biodiversity patterns. Such analyses will be crucial for a better understanding of the exceptionally rich freshwater species radiations found in some regions of the globe. The knowledge emerging from such integrative studies is essential for the recognition and conservation of freshwater biodiversity in a dynamic world.
